# Exosomal MicroRNA: Diagnostic Potential and Role in Breast Cancer Dissemination

**DOI:** 10.3390/molecules30193858

**Published:** 2025-09-23

**Authors:** Svetlana Tamkovich, Alexandra Borisova, Andrey Shevela, Alexander Chernyavskiy, Alyona Chernyshova

**Affiliations:** 1 Institute of Chemical Biology and Fundamental Medicine, Siberian Branch of Russian Academy of Sciences, 630090 Novosibirsk, Russia; a.borisova5@g.nsu.ru (A.B.); chernyshova_a@meshalkin.ru (A.C.); 2 E.N. Meshalkin National Medical Research Center, Ministry of Health of the Russian Federation, 630055 Novosibirsk, Russia

**Keywords:** microRNA, exosomes, EVs, breast cancer, blood, liquid biopsy

## Abstract

Liquid biopsy, which analyzes tumor secretomes in biological fluids, allows us to not only diagnose cancer, but also evaluate the effectiveness of antitumor therapy, predict the prognosis of the disease, and select targeted therapy. One of the promising sources for identifying tumor markers using liquid biopsy is exosomes—small extracellular vesicles (sEVs) (30–150 nm in size) that are secreted by all types of cells, including tumor cells, to exchange information. It is known that during the maturation process, mainly biologically active proteins and non-coding RNA are packaged into exosomes, and tumor cells secrete significantly more exosomes than normal cells. Taking into account the involvement of microRNAs in the mechanisms of carcinogenesis, their high stability in EVs, and ease of detection, exosomal microRNAs are the most promising tumor markers for creating panels that can serve as a guide both for clarifying diagnostics and for making therapeutic decisions on effective cancer treatment, including breast cancer (BC). The purpose of this review is to summarize information on the shortcomings of modern methods for diagnosing early BC, the involvement of exosomal microRNAs in BC dissemination (impact on the immune system, epithelial–mesenchymal transition, proliferation, invasion, migration, angiogenesis, and metastasis), and the high diagnostic potential of exosomal microRNAs for detecting early BC.

## 1. Introduction

Breast cancer (BC) is the most common type of cancer in women. According to the WHO, in 2022, 2,296,840 new cases of BC were registered worldwide, of which 666,103 were fatal [1]. In Russia, BC also ranks first in terms of incidence and mortality among malignant tumors in women aged 40–85 years, with an incidence increase of 13.03% over the past 10 years (from 47.05 in 2013 to 57.28 per 100,000 population in 2023) [2]. The difficulties in BC diagnosis are often due to the asymptomatic course of the disease, as well as the lack of reliable biomarkers that can detect malignant processes in the early stages. It is known that early diagnosis of neoplasms is associated with increased effectiveness of antitumor therapy and a significant reduction in mortality [3]. At the same time, BC diagnosis at late stages correlates with a decrease in five-year survival and an increase in the cost of treatment [4]. Nevertheless, in the Russian Federation in 2022, more than a quarter of new cases of BC were diagnosed at late (III-IV) stages of the disease [5].

The BC diagnosis is made based on physical examination data in combination with imaging methods and confirmed by pathological examination. Imaging methods include bilateral mammography and ultrasound (US) examination of the mammary glands and regional lymph nodes [6]. Magnetic resonance imaging (MRI) is recommended only in cases of familial breast cancer with the presence of a BRCA mutation and suspicion of multifocality.

Mammography, which is the standard diagnostic method, can detect neoplasms measuring 10 mm or more, but its use in dynamic observation is limited by the radiation dose. It has also been shown that mammography can be used to predict the patient’s HER2 status, which can be used for diagnostic and therapeutic purposes [7]. The disadvantages of mammography include age restrictions (increased breast density in patients younger than 40 years), a relatively high frequency of false-negative results in the examination of palpable tumors (up to 15% [6]), and a significant frequency of false-positive results (up to 61% in women aged 40–50 years [8]). To reduce the frequency of false-positive results, mammography is used in conjunction with other imaging methods [9].

US imaging is widely used in the diagnosis of malignant breast tumors. Due to the absence of ionizing radiation, US is safe for examining pregnant and breastfeeding women. Other advantages of this method include its accessibility and higher sensitivity in relation to examinations of denser breast tissue. The limitations of ultrasound include the inability to detect microcalcifications [10] and a significant decrease in sensitivity with a high content of fatty tissue in the mammary gland [11] and the dependence of the results of data interpretation on the qualifications of the physician.

MRI and positron emission tomography (PET) are not subject to the above limitations and provide an accurate assessment of the size and location of lesions with a diameter of 10 mm or more, as well as visualization of microcalcifications and residual malignant foci in patients who have undergone surgery [12]. However, these methods are not used for population screening [11]. PET is an effective method for the instrumental diagnosis of BC. However, in addition to the limitations inherent in MRI, PET is characterized by its inability to detect lesions smaller than 10 mm in diameter [11].

Another modern and relatively accessible diagnostic method for studying breast tumors in nuclear medicine is mammoscintigraphy, which has a number of advantages over the above methods for detecting BC. In particular, the degree of accumulation of the radiopharmaceutical (^99m^Tc-MIBI, ^99m^Tc-Technetril, etc.) in the tumor focus does not depend on the density of the glandular tissue, and allows for detecting tumors from 6 mm in size, including those with multicentric growth [12,13]. The sensitivity of the analysis depends on the biological subtype of BC and is generally 82% for palpable (76% for luminal A and 100% for triple-negative and HER2-positive subtypes) [12] and 52% for non-palpable neoplasms [14].

The main method for diagnosing BC is biopsy. The accuracy of biopsy diagnosis depends on the volume of biomaterial, its purity, and the accuracy of tumor sample collection [15]. As a result, the sensitivity and specificity of this method vary between 52% and 93% [16].

The problem of detecting BC in situ and at stage I of the disease is associated with both the asymptomatic course of the disease and the limitations of instrumental methods of analysis and their availability, as well as the lack of reliable tumor markers for diagnosing the disease. Since the probability of successful treatment when detecting malignant neoplasms at stage I reaches 92–98% [17], the search for new markers in the biological fluids of patients for the timely BC detection is one of the urgent tasks of clinical oncology in general, and molecular oncology in particular. When diagnosing BC using traditional methods, it is necessary to take into account the heterogeneity of the disease both within the nosological form and within a specific case of the disease, since the tumor node may contain foci with different levels of expression of estrogen receptors, progesterone, and HER2. In this regard, biopsy of the central part of the neoplasm, traditionally used for diagnostic purposes, does not guarantee the collection of representative material in the case of a heterogeneous tumor [18]. In addition, during the treatment and progression of malignant neoplasms, their biological characteristics may change. In particular, the sensitivity of the tumor to therapy changes due to the loss of receptors in metastatic and recurrent foci, as well as the acquisition of new mutations, while a repeat biopsy is often difficult, and sometimes technically impossible [19].

**Figure 1:**
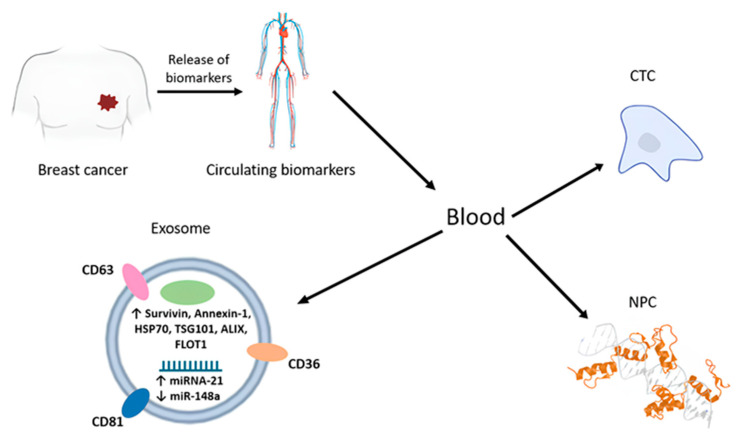
The principle of liquid biopsy and promising tumor markers.

Currently, the detection of circulating tumor cells (CTCs) [19] and extracellular vesicles (EVs), in particular exosomes [20], as well as biopolymers of these cells (nucleic acids [21,22,23,24], proteins [25], and their complexes (NPCs, i.e., nucleoprotein complexes [26,27]) and metabolites [28]) in biological fluids such as blood, urine, tears, saliva, and others, is called liquid biopsy (Figure 1). Currently, an active search is underway for cancer markers that can be used for early and accurate detection and prognosis of tumors using liquid biopsy. For example, it has been shown that the activity of serum proMMP-2 and proMMP-9 (matrix metalloproteinases) in BC patients positively correlates with the size of the tumor, as well as with the presence of metastases in the lymph nodes [29]. At the same time, these markers can also potentially help determine the subtype of BC, since it has been shown that tumor proMMP-2 activity and adjacent tissue aMMP-2 activity are elevated in patients with basal-like carcinoma compared to luminal type A BC [30]. Another marker that may indicate the presence of cancer is lactate dehydrogenase (LDH). It has previously been shown that elevated blood LDH levels can be informative for diagnosing and prognosing the course of the disease, as well as assessing the effectiveness of therapy along with other laboratory tests. Serum LDH is known to be elevated in melanoma, lung cancer, and BC [31], and its activity increases in late stages of lymphoma [32]. It has also been shown that the activity of LDH release from cells in patients with BC negatively correlates with the activity of NK cells [33].

The advantages of using this developed method compared to tissue biopsy include the possibility of conducting an analysis without obtaining tumor tissue, which allows identifying both primary neoplasms and secondary metastatic foci. Another advantage of liquid biopsy is the reflection of the heterogeneity of the neoplasm, since the genetic and phenotypic heterogeneity of the tumor cell population is a serious problem both in establishing the subtype of BC using histological analysis of biopsy material and in treating patients, increasing the likelihood of therapeutic, primarily drug, resistance [34].

Given the higher concentration of exosomes in biological fluids (at least 10^8^ exosomes/mL compared to 10 circulating tumor cells/mL), they appear to be a more promising source of biomarkers for use in liquid biopsy [35]. Furthermore, the molecular cargo of EVs has a number of advantages over NPCs. Firstly, the contents of exosomes are the result of sorting, unlike circulating nucleic acids, which are mainly formed during necrosis and apoptosis [36], which ensures higher specificity of exosomes as biomarkers. Second, the composition of exosomes depends on the composition of the parent cell [37,38], with vesicles containing receptors and adhesion molecules characteristic of the parent cell on their membrane. These surface markers direct exosomes to target cells, which allows us to not only identify the origin of the exosome, but also predict the location of metastases [39]. Thirdly, the lipid membrane of exosomes protects their molecular cargo from hydrolases, ensuring the stability of RNA and proteins over a long period of time, which facilitates the detection of tumor markers compared to tumor DNA, which circulates in the blood in small quantities [40,41]. Fourth, exosomes contain a higher concentration of biomarkers compared to individually circulating molecules in the NPCs [42]. Numerous studies have shown that tumor-derived exosomes promote proliferation, migration, angiogenesis, epithelial–mesenchymal transition (EMT), and resistance to hormone and chemotherapy [43,44]. Thus, liquid biopsy based on the analysis of the composition of exosomes provides more reliable results compared to approaches based on the analysis of nucleic acids in the composition of NPCs [45].

## 2. MicroRNA Biogenesis and Packaging into Exosomes

MicroRNAs are a class of small non-coding RNAs approximately 22 nucleotides in length that participate in post-transcriptional regulation of gene expression by binding to mRNA in the 3′-untranslated region. This interaction can lead to mRNA degradation or inhibition of protein synthesis. Thus, microRNAs play an important role in the regulation of various biological processes: cell proliferation, differentiation, apoptosis, embryonic development, metabolism, etc., since ~60% of human genes are regulated by microRNAs [46].

MicroRNA genes are transcribed into pri-miRNAs, which mature with the help of the Drosha protein complex, forming microRNA precursors (pre-miRNAs). These pre-miRNAs are transported into the cytoplasm through nuclear pores, where they undergo further hydrolysis by the ribonuclease Dicer to form mature microRNAs. The microRNAs are then either packaged into exosomes [47,48] or secreted from the cell into the bloodstream as part of NPCs (e.g., with Ago-2, NPM1) [49] or high-density lipoproteins, which makes them resistant to degradation [50]. Once inside recipient cells as part of exosomes, microRNAs can modulate processes related to proliferation, invasion, metastasis, angiogenesis, and drug resistance [51,52].

The composition and concentration of microRNAs in exosomes differ from those present in the parent cell [53]; moreover, there is a set of microRNAs that are predominantly found in exosomes [54,55]. Although the main mechanism of RNA sorting into exosomes has not yet been fully studied, it is assumed that microRNAs contain specific sequences that are recognized by proteins involved in the sorting of nucleic acids into EVs [55]. To date, several potential pathways for sorting microRNAs into exosomes have been proposed [52,55]:

(1) the neural sphingomyelinase 2 (nSMase2)-dependent pathway;

(2) RNA-binding proteins mediated pathways (AGO2, YBX-1, SYNCRIP, MEX3C, MVP, La protein);

(3) 3′-miRNA sequence-dependent pathway.

Neural sphingomyelinase is a hydrolase involved in the cleavage of sphingolipids into phosphatidylcholine and ceramide, the latter playing a key role in exosome biogenesis [56]. Indirect evidence that nSMase2 is involved in the sorting of microRNAs into exosomes is that inhibition of this enzyme reduces microRNA secretion, while stimulation increases the level of exosomal microRNAs [57,58].

RNA-binding proteins participate in the sorting of specific microRNAs into exosomes. One such protein is sumoylated heterogeneous nuclear ribonucleoprotein (hnRNPA2B1), which specifically recognizes sequences at the 3′-ends of exosomal microRNAs. The sumoylation process, in turn, regulates its binding to microRNAs [59]. Specifically, the GGAG motif (EXO motif) can be recognized by sumoylated hnRNPA2B1 [60], while the GGCU motif (hEXO motif) is recognized by the SYNCRIP protein [61]. This interaction facilitates the selective packaging of certain microRNAs into exosomes [49], indicating that certain sequence elements are crucial for sorting. It is also suggested that the RISC complex may be another regulator of miRNA sorting into exosomes. Phosphorylation of the AGO2 protein, which is part of this complex, is controlled by the KRAS-MEK-ERK signaling pathway and affects the content of miRNAs in exosomes. When AGO2 is knocked out, the content of exosomal microRNAs, such as miR-142-3p, miR-150, and miR-451, decreases [62].

It is assumed that different subpopulations of exosomes [63] may use different mechanisms for sorting microRNAs. For example, some studies have shown selective sorting of RNA in sEVs with high density compared to non-selective sorting in larger vesicles, highlighting the complexity and specificity of microRNA packaging [64]. The packaging of microRNAs into exosomes is also influenced by modification of the 3′-end (uridylation and adenylation), which distinguishes exosomal microRNAs from cellular ones [65].

## 3. Molecular Cargo and Features of Exosome Circulation in the Blood

**Figure 2:**
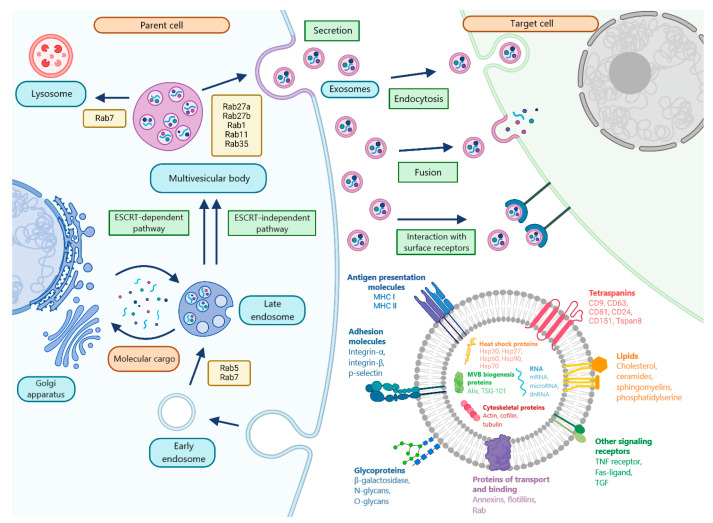
Biogenesis and composition of exosomes.

The formation of exosomes, unlike other types of vesicles, is a multi-step process. In the first stage, invagination of the plasma membrane occurs, resulting in the formation of early endosomes. Next, invagination of the plasma membrane leads to the formation of intraluminal vesicles (ILVs). The subsequent accumulation of multiple ILVs leads to the formation of multivesicular bodies (MVBs), which can be degraded by fusion with lysosomes or autophagosomes, or transported to the plasma membrane and, by fusion with it, release ILVs into the intercellular space in the form of exosomes. Transformation into MVB can occur both with and without the participation of the ESCRT protein complex (Figure 2) [66].

The mechanisms of exosome secretion are dependent on numerous factors, including the type of parent cell and the molecular cargo of the vesicles. It is evident that Rab GTPases, in conjunction with SNAP and SNARE proteins, assume a pivotal function in this process. Furthermore, Rab27a and Rab27b have been shown to promote the fusion of MVB with the plasma membrane, and Rab-1, Rab11, and Rab35 have been identified as regulators of the process of exosome secretion [67]. Exosomes have been demonstrated to interact with target cells through three principal mechanisms: interaction with cell receptors, fusion with the cell membrane, and endocytosis [49].

Regardless of their origin, exosomes contain a universal set of components, such as proteins involved in the formation, secretion, transport, and binding of exosomes to recipient cells, as well as lipids, metabolites, and other components that partially reflect the composition of the parent cells (Figure 2) [68]. Examples of universal exosomal proteins include tetraspanins (CD9, CD63 and CD81), adhesion receptors, annexins, integrins and flotillins. These proteins play a role in the processes of recognition and binding to the recipient cell [68,69,70]. MMPs, heat shock matrix proteins (Hsp70 and Hsp90), and proteins involved in biogenesis (Alix and TSG 101) are also considered to be universal exosomal proteins.

It has been established that enzymes comprise approximately one-third of exosome proteins. MMPs have been shown to belong to tetraspanin-associated proteases [71]. The process of ADAM-mediated hydrolysis of the extracellular domains of transmembrane proteins has been shown to regulate a number of cellular processes, including cell adhesion, migration, and intercellular interactions [72]. MMPs have been shown to hydrolyze type IV collagen, elastin, fibronectin and laminin, as well as cell surface proteins, including E-cadherin, fibrin and IL-1 [72]. MMP-2 and MMP-9 are the most abundant MMPs in exosomes [73,74]. As demonstrated in the extant literature, there is evidence to suggest that ADAM-10 levels increase in blood exosomes in BC [75,76]. However, the levels of other tetraspanin-associated proteases in exosomes in this disease remain to be elucidated.

Tetraspanin non-associated proteases include PAPP-A and 20S proteasomes. The former is a metalloproteinase [77], while the *β*-subunits of the latter exhibit caspase-like, trypsin-like, and chymotrypsin-like activity [78]. As demonstrated in the extant literature, there is an elevated level of 20S proteasome observed in patients diagnosed with BC, both in tumor tissue and in the circulating exosome form found in blood [79].

Various types of RNA have been identified in exosomes, including microRNA, messenger RNA (mRNA), ribosomal RNA (rRNA), long non-coding RNA (lncRNA), circular RNA, and others [80].

lncRNAs are a class of non-coding RNA molecules longer than 200 nucleotides that participate in the regulation of gene expression. These RNAs are capable of modulating translation processes, ensuring protein stability, and controlling mRNA translocation [81]. It has been established that lncRNAs play a key role in the development of various types of malignant neoplasms, including BC. For example, the HOTAIR miRNA is involved in invasion, metastasis, and tumor growth in various types of cancer, including BC; a positive correlation has also been found between this miRNA in exosomes and the expression level of the ERBB2 (HER-2/Neu) gene [82]. Some lncRNAs have oncogenic activity, while others exhibit tumor-suppressive properties. Differential lncRNA expression significantly influences the development of primary and acquired resistance to radiation, endocrine, immune and targeted therapies [83]. High levels of MIR100HG, T376626, and XIST microRNAs are associated with an unfavorable prognosis in triple-negative BC (TNBC) [84,85], while suppression of MIR100HG expression significantly reduces the proliferation of MDA-MB-231 cells [86]. It has also been shown that SNHG1 miRNA participates in the modulation of tumor-associated M2 macrophage polarization, thereby stimulating BC progression [85,86].

Currently, the role of circular RNAs in the BC dissemination is also being actively researched. Despite an incomplete understanding of the functions of these RNAs, it is assumed that they are involved in tumor formation, progression and metastasis through interaction with microRNAs and proteins [87]. Circular RNAs are considered as possible tumor markers, partly due to their stability, which is acquired through their non-linear shape. It has also been shown that there are exosomal sorting mechanisms for these RNAs, which are likely related to the level of microRNA targets in the parent cell [88]. In vitro and in vivo studies have shown that the levels of some circular RNAs differ between healthy donors and BC patients [89] and also depend on the degree of tumor malignancy, with these observations being characteristic of both blood samples and exosomes [90].

Exosomal tumor-derived mRNA has been demonstrated to exert influence on a variety of processes in recipient cells [91]. It has been demonstrated that MCF-7 exosomes contain hTERT mRNA, which can be transcribed into active telomerase, thus promoting oncogenic processes [92], while exosomal WNT5a mRNA from the MCF-7 BC cell line promotes invasion by activating the β-catenin-independent WNT-signaling pathway in macrophages [93].

## 4. The Role of Exosomal MicroRNA in BC Dissemination

**Figure 3:**
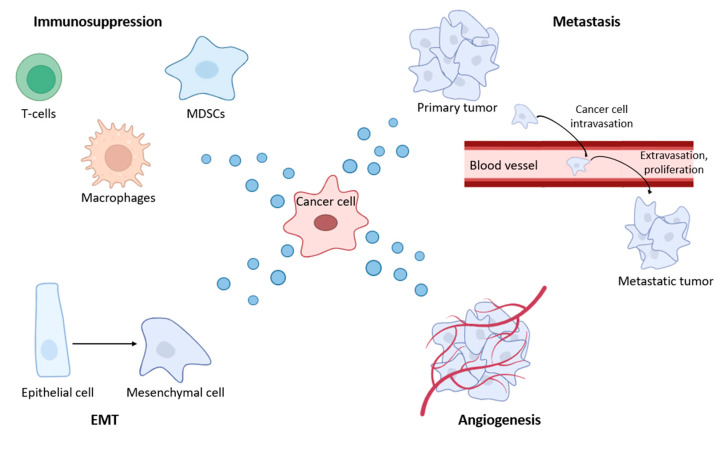
The role of exosomes in BC dissemination.

Exosomes play a key role in regulating various biological processes, both normal and pathological, including cancer (Figure 3). Their influence spans embryogenesis, immune response, tissue regeneration, hemostasis, angiogenesis, etc. [94].

Despite the rapid growth in the number of scientific publications devoted to the study of exosomes over the past ten years, many questions remain in understanding the regulation of intercellular communication through exosomes. This concerns, in particular, the dynamics and regulation of exosome secretion by cells, the mechanisms of intracellular sorting of molecules in exosomes, and the targeted transport of their contents between cells.

### 4.1. The Influence of BC Exosomes on the Functional Activity of the Immune System

BC-derived exosomes have been demonstrated to play a pivotal role in the formation of a microenvironment with a less active immune system within the tumor, thereby exerting an immunosuppressive effect. One way in which tumor-derived exosomes influence the immune system is by stimulating the polarization of tumor-associated M2 macrophages (TAMs), which regulate all stages of tumor progression, including angiogenesis, immunosuppression, drug resistance and metastasis in BC [95,96]. In particular, the role of tumor-derived exosomes in macrophage polarization has been demonstrated in cell lines mimicking TNBC [97]. It has been found that exosomal miR-222 also participates in the stimulation of TAM polarization in adriamycin resistance [98]. By influencing the phenotype of macrophages, microRNAs indirectly affect other elements of the immune system. It has been shown that under conditions of endoplasmic reticulum stress, the content of miR-27a-3p is increased in the exosomes of BC cells, which indirectly stimulates macrophage polarization and contributes to an increase in the frequency of T cell apoptosis [99].

The molecular cargo of exosomes can affect not only macrophages but also other cells of the immune system. For example, in BC, miR-9 and miR-181a in tumor-derived exosomes stimulated the development of myeloid-derived suppressor cells, which suppress the immune response [100].

Furthermore, exosomal microRNAs have been demonstrated to elicit an immunomodulatory effect, which can be exploited for therapeutic purposes. For instance, the administration of exosomes containing Let-7i, miR-142 and miR-155 to mice with BC had an effect on T-cell activation and dendritic cell maturation [101]. In addition, let-7 likely prevents the establishment of immunosuppressive pre-metastatic niches in BC, as the occurrence of an immunosuppressive effect is contingent upon the low content of this microRNA in exosomes, concomitant with the transition of neutrophils to a pro-tumor phenotype [102].

Consequently, the immunosuppressive microenvironment formed is pivotal in enabling the tumor to evade immune surveillance. The comprehension of these mechanisms has the potential to facilitate the development of novel immunotherapeutic approaches that are aimed at restoring the antitumor immune response and overcoming resistance to therapy.

### 4.2. BC-Derived Exosomes Stimulate EMT, Proliferation, Invasion and Migration

It is currently known that the molecular cargo of exosomes, including microRNAs, is involved in all stages of the EMT, up to the formation of metastases [103,104].

In vitro and in vivo studies show that exosomal miR-181d-5p promotes EMT by reducing the levels of the transcription factors CDX2 and HOXA5 [105]. A similar study found that miR-191–5p levels are elevated in both BC cell lines and blood exosomes from BC patients, and that it stimulates EMT by negatively regulating KLF6 [106]. In the MCF-7 cell line, it has been shown that cancer stem cells can mediate EMT and chemoresistance, most likely through exosomal transport of miR-155 [107]. Another in vitro study revealed the role of miR-197 cancer stem cell EVs in EMT [108].

EMT is also stimulated by exosomes from cells in the tumor microenvironment, such as cancer-associated fibroblasts (CAFs). In BC cell lines, it has been shown that miR-21, -378e, and -143 in CAF exosomes promote EMT, tumor cell growth, and the development of an aggressive tumor phenotype [109]. At the same time, it has been shown that the activation of normal fibroblasts is induced by miR-370-3p in the exosomes of BC cells, contributing to increased migration, invasion, and EMT of cancer cells [110].

MicroRNAs in the exosomes of BC cell lines are also involved in stimulating the invasion and migration of tumor cells. For example, MCF-7 and MDA-MB231 cells show increased levels of exosomal miR-301a, miR-106b, and miR-328, which are involved in the processes of increased cell migration and invasion [111]. Incubation of exosomes containing miR-222 with various BC cell lines promoted cell migration and invasion by suppressing PDLIM2 expression [112]. Furthermore, studies have demonstrated that exosomal miR-10b functions as a tumor suppressor by reducing the protein levels of its target genes, including HOXD 10 and KLF4, and by enhancing the invasive capacity of BC cells [113]. Additionally, exosomal miR-105 has been shown to regulate cell migration by targeting ZO-1 [114]. In addition to stimulating migration and invasion, microRNAs in exosomes can also suppress these processes. For example, a study of the role of histone demethylase LSD1 in BC found that knockdown of this gene leads to a decrease in the level of miR-1290 secreted in exosomes, which stimulates EMT, migration, and invasion of BC cells [115]. Furthermore, studies have demonstrated that miR-5100 exerts a regulatory effect on invasion, migration, and EMT in both pancreatic cancer and BC [116].

Exosomal non-coding RNAs not only stimulate cell invasion and migration, but also influence cell proliferation. For example, it has been shown that exosomal miR-130a and miR-425 increase the viability of MCF-7 cells, whose proliferation is associated with signaling pathways such as TOR, ErbB, MAPK and TGF-β [117]. Incubation of exosomes from the culture medium of the HCC1806 line, which mimics TNBC, with the non-carcinogenic MCF10A line caused an increase in cell proliferation, which was probably due to changes in 138 genes and the expression of 70 microRNAs affecting PI3K/AKT, MAPK, and HIF1A [118]. It has been shown that in BC, the level of exosomal miR-222 is significantly increased in the plasma of patients in the late stages of the disease and correlates with the high aggressiveness of BC cell lines [119].

### 4.3. BC-Derived Exosomes Stimulate Angiogenesis and Metastasis

Exosomes of tumor origin play an important role in regulating angiogenesis accompanying tumor development. In particular, MMPs participate in creating conditions for capillary growth by degrading the basal membrane, as well as in the release and induction of transcription of angiogenic factors [116].

In addition, it is known that exosomal microRNAs also play a role in angiogenesis in BC. Members of the miR-17-92 cluster, for example, have a proangiogenic effect by targeting thrombospondin 1, VEGFA, and TIMP. In particular, miR-20a promotes the expansion of the vascular network in BC, probably mediating this effect through VEGFA [120]. When studying the effect of intracellular calcium ion concentration on angiogenesis, an increase in miR-145 levels was found in exosomes obtained from BC cells with reduced calcium concentration. Treatment of HUVECs with exosomes from these cells reduced their ability to undergo angiogenesis. This effect is likely due to the influence of miR-145 on IRS1 [121]. It is also suggested that increased levels of miR-92a in both plasma exosomes and total blood exosomes of BC patients lead to a proangiogenic effect [43], which has also been demonstrated in other neoplasms [122,123].

It is evident that tumor cells are pivotal in the process of metastasis, as evidenced by their ability to form metastatic niches and stimulate the formation of primary metastatic nodes. For instance, exosomal miR-122 has been demonstrated to promote the formation of metastatic niches by altering glucose consumption in recipient cells [124]. In addition, it has been demonstrated that exosomes with elevated levels of miR-200 have the capacity to enhance the metastatic potential of BC cells in both in vivo and in vitro contexts [125].

Tumor cells are characterized by metastasis to specific organs with the formation of organotropic metastases. At the initial stage, the interaction between tumor cells and target organs leads to the formation of pre-metastatic niches. This process is then reinforced by other intercellular interactions, including tumor cell exosomes. In particular, the study showed that exosome secretion by T47D-CXCR4 cells is enriched in mRNA associated with stem cells and metastasis, confirming the predisposition of breast cancer cells to metastasis and the role of exosomes in stimulating tumor progression and metastasis [126].

TNBC is characterized by an increased frequency of metastasis to the lungs and brain, which distinguishes it from other molecular subtypes, for which metastatic involvement of bones and soft tissues is more typical. Numerous studies have demonstrated the upregulation of microRNAs, such as miR-9, miR-20a-5p, and miR-155, in highly metastatic TNBC cells and tumor-derived exosomes. Transfer of miR-20a-5p by exosomes to bone marrow macrophages was shown to stimulate osteoclastogenesis [127,128]. Studies show that elevated levels of exosomal miR-105 are characteristic of BC with high metastatic potential [129]. A number of other studies have identified a significant correlation between elevated levels of exosomal miR-210 and the presence of metastases in the lymph nodes in patients diagnosed with BC. The secretion of this microRNA is regulated by nSMase2, which promotes the metastasis of cancer cells by inducing angiogenesis in the tumor microenvironment, enhancing endothelial cell migration and capillary formation [56]. In addition, it has been demonstrated that cancer exosomes play a role in the disruption of the blood–brain barrier, which may lead to the formation of brain metastases. One of the putative mediators of this process is miR-181c, which alters actin localization [130].

The role of exosomal miR-210 as one of the key factors of tumor angiogenesis and brain metastasis was also noted, with high expression of miR-210 correlating with poor survival in BC patients with brain metastases [131]. Studies conducted on cell lines mimicking HER2+ BC have shown that miR-146a-5p not only promotes resistance to trastuzumab but also stimulates angiogenesis, proliferation, and migration [132]. Some microRNAs, such as miR-218, are known to act as suppressors of angiogenesis, migration, and metastasis [133]. Another example of a tumor suppressor is miR-124-3p, which targets the MTDH oncogene [134]. In vitro, downregulation of miR-145 has been shown to induce angiogenesis. Presumably, this effect is observed due to the fact that IRS1 is no longer regulated by miR-145 [122].

Therefore, the role of exosomes as important messengers in intercellular communication is significant both within the primary breast tumor microenvironment and in metastatic niches.

## 5. Exosomal MicroRNAs as Markers for BC Detection

Since exosomes mainly contain RNA less than 200 nucleotides in size [135], microRNA attracts the most attention from researchers. In particular, on 11 July 2025, a search of the PubMed database for the combination ‘microRNA and exosome and cancer’ yielded 4978 articles, while a search for ‘exosomes and microRNA and breast cancer’ yielded 608 articles.

To date, according to the ExoCarta database, 2838 microRNAs have been identified in the molecular cargo of exosomes. Although it is still unclear which microRNAs are involved in carcinogenesis, the level of tissue-specific microRNAs in exosomes can be used to develop diagnostics for malignant neoplasms, including BC.

In 2016, Exosome Diagnostics launched the first ExoDX test on the US market, which allows assessing the advantages of prostate cancer liquid biopsy using ERG, PCA3, and SPDEF mRNA in urine exosomes [136]. This test currently has Breakthrough Device Designation status from the FDA, but has not yet been approved for widespread use in clinical practice. It is imperative to recognize that 20–25% of women diagnosed with localized BC receive neoadjuvant therapy as their initial treatment line. Consequently, the identification of effective biomarkers in liquid biopsy for the diagnosis and prognosis of treatment response is a pressing task in the field of molecular oncology. A comparative analysis of CTCs and exosomal microRNAs in blood samples before and after neoadjuvant therapy showed that elevated levels of exosomal miRNA-21, miRNA-222 and miRNA-155 significantly correlate with the presence of CTCs, indicating the potential role of both exosomal microRNAs and CTCs in improving diagnosis and prognosis for patients undergoing neoadjuvant therapy [137].

A promising biomarker for the diagnosis and prognosis of BC may be the level of exosomal miR-148a in the blood, which is significantly reduced in patients with BC and correlates with an unfavorable prognosis [138]. Furthermore, the level of exosomal miR-223-3p has been shown to have potential as an additional tool in the diagnostic toolkit for BC, with a statistically significant increase in its concentration in plasma exosome samples observed in patients with ductal carcinoma of the breast, even at the initial stage of the disease [139]. In addition, exosomes associated with the surface of blood cells may serve as a more accurate source of tumor markers, in particular microRNAs [140].

The combination of exosomal microRNAs with other clinical biomarkers significantly improves the analytical characteristics of diagnostic systems based on liquid biopsy. For example, a panel of four exosomal microRNAs (miR-424, miR-423, miR-660, and let7-i) can differentiate between healthy donors and BC patients with 98.6% sensitivity and 100% specificity [141]. It is noteworthy that in this study, urine exosomes served as a probable source of tumor markers.

Using NGS (Next Generation sequencing) of serum exosomal microRNAs and subsequent verification by real-time PCR (polymerase chain reaction), a highly effective panel of markers has been developed for the differential diagnosis of breast neoplasms: the combination of miR-142-5p and miR-320a allows the luminal A subtype to be distinguished from the TNBC with 100% sensitivity and 93.8% specificity [142]. In addition, a diagnostic panel based on the analysis of miR-195, miR-210, miR-21, and miR-16 has a sensitivity of 71.4% and a specificity of 100% for BC diagnosis [143]. The potential of erythrocyte exosomes as sources of tumor markers, including for BC, is also being studied [144].

Despite the promising potential of miRNAs, the implementation of EV-based liquid biopsy into clinical practice is associated with several challenges. First, there is currently no EV isolation method available and scalable for use in a healthcare setting, guaranteeing high purity and the ability to simultaneously analyze large numbers of samples. Methods used for EV isolation, such as ultracentrifugation, density gradient centrifugation, and polymer precipitation, are time-consuming and may not be suitable for routine analysis. Methods based on immunospecific binding of EVs to a carrier are expensive, which is also a serious limitation for implementation into clinical practice [145]. Second, there are currently no standardized approaches to quantification and normalization of vesicular miRNAs. To date, researchers have used various strategies for miRNA level normalization, including the use of endogenous controls, exogenous controls, mean normalization, and paired miRNA normalization, which often do not allow comparison of data obtained by different groups [146,147,148,149].

## 6. Exosomal MicroRNAs Associated with BC Relapse

To identify key molecular mechanisms underlying BC recurrence, a bioinformatics analysis was performed. Based on the analysis of literature data, sixteen exosomal microRNAs were selected, the level of which changes depending on the presence or absence of relapse and pathological complete response (pCR) to therapy, the latter of which is associated with a more favorable prognosis for BC patients (Table 1).

The target genes of the selected microRNAs were initially identified using the DIANA-miRPath v4.0 database (http://62.217.122.229:3838/app/miRPathv4). DIANA-miRPath enables the identification of KEGG (Kyoto Encyclopedia of Genes and Genomes) and GO (Gene Ontology) biological pathways that are enriched with target genes for specific microRNAs. This allows us to determine the potential influence of these microRNAs on cellular processes associated with BC recurrence. The results show that these microRNAs are involved in BC pathways and other cancer pathways, as well as in cell cycle and apoptosis-related processes and in mTOR, FoxO and p53 signaling pathways. All studied miRNAs, except miR-548ab, are involved in BC-related pathways and participate in the regulation of the expression of 89 target genes.

**Figure 4:**
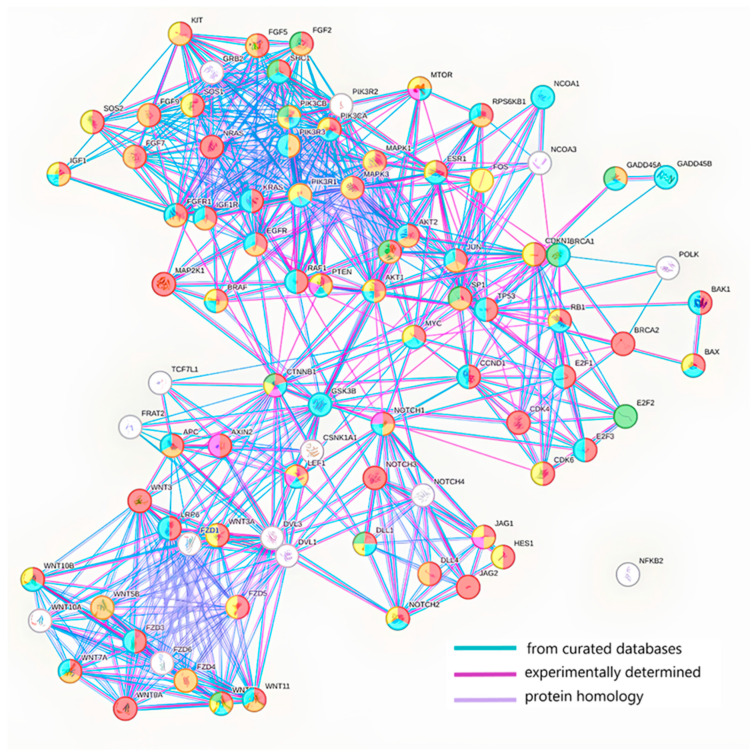
Network of interactions between target genes involved in BC. Regulation of cell population proliferation—red color; regulation of apoptotic processes—blue; regulation of EMT—pink color; regulation of cell migration—orange color; regulation of angiogenesis—green color; regulation of immune system processes—yellow color.

The STRING v12.0 database (string-db.org) was used to analyze interactions between the protein products of target genes. STRING provides information on known and predicted protein interactions, including physical interactions, protein homology and participation in common biological pathways. Using target genes identified in DIANA-miRPath, an interaction network was constructed to identify key proteins that may play a central role in regulating the processes leading to BC recurrence. The analysis revealed that these proteins are involved in regulating proliferation, apoptosis, cell migration, EMT, immune processes and angiogenesis (Figure 4).

The obtained protein interaction network was imported into the Cytoscape software, version 3.10.3 (cytoscape.org) to identify hub proteins. Hub proteins were identified based on degree centrality analysis, which was calculated using Cytoscape’s built-in NetworkAnalyzer tool. Degree Centrality determines the number of direct interactions a protein has with other proteins in the network, so proteins with the highest number of interactions were identified as hub proteins. Network topology analysis, particularly Degree Centrality, enabled us to identify key proteins that may play a central role in regulating processes that lead to breast cancer recurrence. The degree value ranged from 26 to 0, with 17 proteins having the highest values (21–26): PIK3R1, CTNNB1, KRAS, MAPK1, PIK3R2, PIK3CA, MAPK3, EGFR, AKT1, TP53, PIK3CB, NRAS, GSK3B, SHC1, DVL1, GRB2 and DVL3. These results highlight the significant role of these proteins in dissemination and, in particular, BC recurrence.

Thus, the bioinformatics analysis revealed several miRNAs, associated genes, biological pathways and protein interactions that are potentially involved in BC recurrence. The identified protein hubs and biological pathways are promising targets for further research into developing new strategies for preventing and treating BC recurrence. Further experimental confirmation of the role of these microRNAs and their targets will allow the creation of panels for assessing the prognosis of the course and selection of targeted therapy for BC.

## 7. Conclusions

Modern instrumental methods of BC diagnostics have a number of limitations related to early detection of the disease and differentiation of benign and malignant tumors. Rapid development of molecular oncology opens up prospects for the emergence of new tumor markers in the next five years, including those based on exosomal microRNAs for the detection of early BC. In combination with instrumental diagnostic methods, these markers will allow more effective detection of BC and assessment of the disease prognosis. Elucidation of detailed mechanisms of tumor spread involving exosomal microRNAs will allow the development of gene-directed therapeutic approaches to increase the effectiveness of BC treatment. This, in turn, will lead to a decrease in disability and improvement in the quality of life of women suffering from this disease.

## Figures and Tables

**Table 1:** MicroRNAs associated with BC relapse and pCR.

#	MicroRNAs	Level Change
1	miR-338-3p	↑ in serum exosomes of patients with relapse compared to patients without relapse [150]
2	miR-340-5p	
3	miR-124-3p	
4	miR-29b-3p	↓ in serum exosomes of patients with relapse compared to patients without relapse [150]
5	miR-20b-5p	
6	miR-17-5p	
7	miR-130a-3p	
8	miR-18a-5p	
9	miR-195-5p	↓ in serum exosomes of patients with relapse compared to patients without relapse [150] ↑ in serum exosomes of non-pCR patients with relapse compared to those without it [151]
10	miR-486-5p	↓ in serum exosomes of patients with relapse compared to patients without relapse [150]
11	miR-93-5p	
12	miR-548ab	↓ in serum exosomes of non-pCR patients with relapse compared to those without it [151]
13	miR-155	↓ plasma exosomes after therapy, associated with pCR [152]
14	miR-301	
15	miR-30b	↓ in plasma exosomes of patients with relapse compared with primary BC patients [153]
16	miR-16	↑ in plasma exosomes of patients with relapse compared with healthy donors [153]

## Abbreviations

The following abbreviations are used in this manuscript:

BC	Breast cancer
CAF	Cancer-associated fibroblasts
CTC	Circulating tumor cells
EMT	Epithelial–mesenchymal transition
GO	Gene Ontology
ILV	Intraluminal vesicles
KEGG	Kyoto Encyclopedia of Genes and Genomes
LDH	Lactate dehydrogenase
MMPs	Matrix metalloproteinases
MRI	Magnetic resonance imaging
MVB	Multivesicular body
NGS	Next Generation sequencing
NPC	Nucleoprotein complex
pCR	Pathological complete response
PCR	Polymerase chain reaction
PET	Positron emission tomography
RNA	Ribonucleic acid
sEVs	Small extracellular vesicles
TAM	Tumor-associated macrophages
TNBC	Triple-negative breast cancer
US	Ultrasound examination
WHO	World Health Organization
